# Supplementary material for: Chromothripsis during telomere crisis is independent of NHEJ, and consistent with a replicative origin
Source: Genome Res. 2019 May;29(5):737–49. doi: 10.1101/gr.240705.118 (PMC6499312; doi:10.1101/gr.240705.118)
Supplement: Supplemental Material [file supp_gr.240705.118_Supplemental_file_1.zip › contigs/annotated_contigs/DB39/contig.2.DB39_length_602_mean_cov_5.90365448505.docx]

**DB39_length_602_mean_cov_5.90365448505**

```
CGAAATACAAATCAAAATACATTTTTGTTACATATCATTTTAACAAGCTTAACATTCTTTTCAATACAAGATGACTCAATATAGGTATT
 >chr3:186481342-186481586 - E=4e-135 p=0e+00
TTTAAAAAGCCATGCATTTTATTTTAGAGTAAACAGCTCATCACAAAAATACGTGGGTACATGGGCTATTGTTGTTTACTATTTCAGAG

CACACCCTCCAACTTAACATTTGGTCCACTAAAATATGTAAATGTCCGTCTGGGCACGGTGGCTCA|GATCTTTTCAAGATCTTTTCAA

GAAAACTACTGGCTCAGATCTTTTCAAGATGTCATTCTCAGCTCATCTTCATCTCATTAGGTTAAAGAAGTCCTCTAGATGTTGGGAAA
 >chr3:186598587-186598923 - E=2e-187
GTCAAGACGCACGAGACAGGCTTCCTGACAATGGTAGCTAACGCTCTGGCACTCAGAGAGGGCAAATCAGATGCACCAGGGCCTCTTCT

ACTTGTTAAAACTACATCTTACACCATGTTGCTTCCTATTATAACAGTAGCTGTTGCAATTTTGTTCATTCTGCCTCATATTGCAACAA

AGAAGATTCTGGAGTAGCTTTTTTCTGTTGGCGCTTATTTAGAGTGGAGGAAAATAATGAAAGCCATCG
```
